# Generation of a neuro-specific microarray reveals novel differentially expressed noncoding RNAs in mouse models for neurodegenerative diseases

**DOI:** 10.1261/rna.047225.114

**Published:** 2014-12

**Authors:** Ronald Gstir, Simon Schafferer, Marcel Scheideler, Matthias Misslinger, Matthias Griehl, Nina Daschil, Christian Humpel, Gerald J. Obermair, Claudia Schmuckermair, Joerg Striessnig, Bernhard E. Flucher, Alexander Hüttenhofer

**Affiliations:** 1 Division of Genomics and RNomics, Innsbruck Biocenter, Medical University of Innsbruck, 6020 Innsbruck, Austria; 2 RNA Biology Group, Institute for Genomics and Bioinformatics, Graz University of Technology, 8010 Graz, Austria; 3 Department of Psychiatry and Psychotherapy, University Clinic of General and Social Psychiatry, Innsbruck Medical University, 6020 Innsbruck, Austria; 4 Division of Physiology, Department of Physiology and Medical Physics, Innsbruck Medical University, 6020 Innsbruck, Austria; 5 Pharmacology and Toxicology, Institute of Pharmacy, and Center for Molecular Biosciences, University of Innsbruck, 6020 Innsbruck, Austria

**Keywords:** noncoding RNAs, snoRNA, microarray, Alzheimer's disease, Parkinson's disease, voltage-gated Ca^2+^ channels

## Abstract

We have generated a novel, neuro-specific ncRNA microarray, covering 1472 ncRNA species, to investigate their expression in different mouse models for central nervous system diseases. Thereby, we analyzed ncRNA expression in two mouse models with impaired calcium channel activity, implicated in Epilepsy or Parkinson's disease, respectively, as well as in a mouse model mimicking pathophysiological aspects of Alzheimer's disease. We identified well over a hundred differentially expressed ncRNAs, either from known classes of ncRNAs, such as miRNAs or snoRNAs or which represented entirely novel ncRNA species. Several differentially expressed ncRNAs in the calcium channel mouse models were assigned as miRNAs and target genes involved in calcium signaling, thus suggesting feedback regulation of miRNAs by calcium signaling. In the Alzheimer mouse model, we identified two snoRNAs, whose expression was deregulated prior to amyloid plaque formation. Interestingly, the presence of snoRNAs could be detected in cerebral spine fluid samples in humans, thus potentially serving as early diagnostic markers for Alzheimer's disease. In addition to known ncRNAs species, we also identified 63 differentially expressed, entirely novel ncRNA candidates, located in intronic or intergenic regions of the mouse genome, genomic locations, which previously have been shown to harbor the majority of functional ncRNAs.

## INTRODUCTION

The central nervous system (CNS) contains a large number of different cell types, which express 80% of all protein-coding genes from the human genome, thus exceeding gene expression in all other organs (Lein et al. 2007). Thereby, gene expression is highly diverse in the different cell types due to transcriptional as well as post-transcriptional regulation by proteins, and also changes during development and upon environmental stimuli.

In general, nonprotein-coding RNAs (ncRNAs) add a second layer of complexity to the regulation of gene expression in all human tissues, including the CNS. In contrast to protein-coding genes whose numbers are comparable in higher eukaryal genomes, the number of ncRNA genes is predicted to positively correlate with the developmental complexity of the respective organism (Lein et al. 2007). Thus, it has been speculated, that ncRNAs might regulate development and consequently also brain function in humans (Mattick 2011).

While ∼75% of the human genome is transcribed into RNA, the majority of these RNA transcripts lack protein-coding potential (Djebali et al. 2012) and thus might represent regulatory ncRNAs (Birney et al. 2007; Washietl et al. 2007). In the past, various ncRNA species have been shown to exhibit essential functions in the regulation of gene expression, thereby also playing key roles in neural development, neural plasticity, and brain aging (Mattick 2011). In addition, specific ncRNAs have also been implicated in a number of CNS diseases (Cavaille et al. 2000; Kim et al. 2007; Stark et al. 2008; Taft et al. 2009a, 2010; Haramati et al. 2010; Hebert et al. 2010). MicroRNAs (miRNAs), sized ∼21–23 nt, represent a well-characterized class of small ncRNA molecules with essential roles in CNS development and disease. For expression analysis of miRNAs, numerous commercial tools, such as microarrays or qPCR-panels, have been applied to screen human patient samples as well as animal disease models (Delay et al. 2012; Mouradian 2012). Upon identification of differentially expressed miRNAs, computational tools are readily available to identify their potential target genes and thus elucidate their function. Thus, by these approaches several miRNAs have been implicated in neurological diseases (Cox et al. 2010; Edbauer et al. 2010; Alexandrov et al. 2012; Gardiner et al. 2012; Geekiyanage et al. 2012; Haghikia et al. 2012).

In contrast, much less is known about the biological function and differential expression of non-miRNAs in the CNS, including piRNAs, snoRNAs, or snRNAs, respectively, as well as currently unclassified ncRNAs which are either independently transcribed or which can also be processed from known classes of ncRNAs (Ender et al. 2008; Lee et al. 2009; Taft et al. 2009b; Li et al. 2012). Indeed, there is strong evidence for the participation of non-miRNA species in the etiology of neurodevelopmental diseases. Notably, snoRNA HBII-85 (also designated as SNORD116), located on chromosome 15, has been reported to be implicated in the Prader–Willi syndrome (PWS) (Cavaille et al. 2000; Sahoo et al. 2008), while a snoRNA cluster mapping to chromosome 14q32.3 has been shown to be involved in uni-parental disomy (Bachellerie et al. 2002).

Hence, in this study we focused on expression profiling of short and medium-sized ncRNAs, ranging from 18 to 400 nt in size, in mouse models for CNS diseases. Since up till now, no microarrays have been available to profile expression of these non-miRNA species, we have developed a novel screening platform by using a customized microarray technology through preselection and enrichment of functional ncRNAs from the CNS. Although >400,000 ncRNA candidates have been predicted in the human genome (Hannon et al. 2006), the number of truly functional ncRNAs is currently debated (Brosius 2005; Huttenhofer et al. 2005). In order to enrich for functional ncRNA candidates on the microarray platform, we selected novel ncRNAs from neural or neuronal tissues, respectively.

Here, in contrast to miRNA chips, we describe the successful generation of a custom-made neuro-ncRNA microarray for a highly heterogeneous group of novel neural ncRNAs, exhibiting different sizes, sequences and hence secondary/tertiary structures, which have largely been uncharacterized, up till now. For proof of principle, we investigated ncRNA expression upon changes in calcium channel (Ca_V_) activity, which has been implicated in a variety of neurological disorders such as psychiatric disorders (O'Roak et al. 2012; Cross-Disorder Group of the Psychiatric Genomics Consortium 2013) or Parkinson's disease, respectively (Sulzer and Surmeier 2013). Thereby, we analyzed differential expression of ncRNAs in knockout mouse models for one of the two brain L-type calcium channels (Ca_v_1.3) being implicated in Parkinson's disease (Sulzer and Surmeier 2013) and in lethargic mice, which harbor a nonsense mutation in the gene encoding for the auxiliary voltage-dependent Ca^2+^ channel β_4_ subunit (Cacnb4), previously described as a model for idiopathic epilepsy and ataxia (Burgess et al. 1997; Tadmouri et al. 2012). In addition, we applied the customized neuro-ncRNA microarray to a well-characterized triple-transgenic mouse model (3xTG) for Alzheimer's disease (Oddo et al. 2003).

## RESULTS AND DISCUSSION

### Generation of a custom neuro-ncRNA microarray

In this study, we have developed a novel custom neuro-ncRNA microarray for expression profiling of small and medium-sized ncRNA species (i.e., sized from 18 to 400 nt) in mouse models for CNS diseases, which might be also applicable for other tissues as well as human samples (see below). In a first step, we aimed to identity a comprehensive collection of functional neural or neuronal small ncRNAs. To that end, all sequences used for microarray design were selected from RNA-Seq data, which we previously obtained from (a) murine embryonic stem cells (ES cells) differentiated into neural cells, resulting in three successive stages of differentiation (Skreka et al. 2012), (b) whole mouse brain (Rederstorff et al. 2010), and (c) dorsal root ganglia from mouse embryos as well as adult mice (K Skreka, C Bandtlow, G Dechant, M Rederstorff, and A Hüttenhofer, in prep.).

In contrast to all other approaches aiming for the identification of novel ncRNAs, we isolated RNA-protein particles (RNPs) prior to RNA-seq analysis, a method previously reported by our laboratory (Rederstorff et al. 2010; Rederstorff and Huttenhofer 2011). Since in Eukarya, all functional ncRNAs have been shown to be associated with RNA binding proteins, thereby forming RNPs, by this approach we enriched for biologically functional ncRNAs. These analyses resulted in selection of 1213 ncRNA candidates from the above data set for microarray design.

**FIGURE 1.**
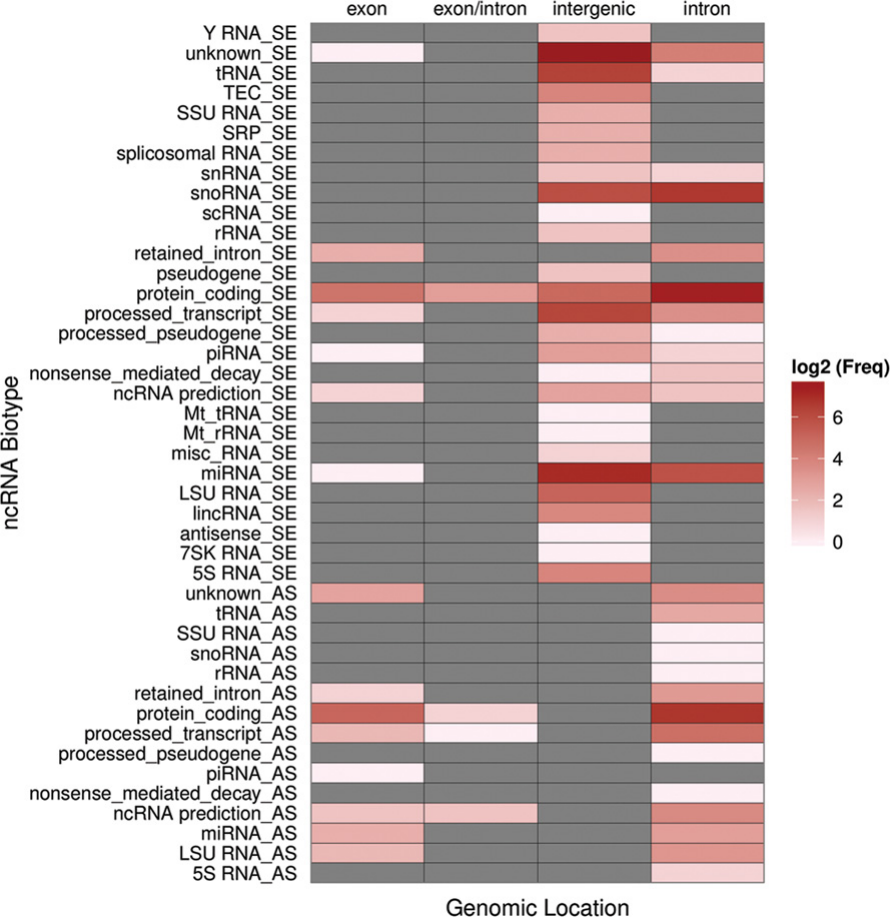
Biotype and genome location of ncRNAs from the neuro-ncRNA microarray. ncRNAs are sorted by ncRNA biotype and genomic locations. Frequencies are illustrated in log_2_ scale. Frequency of zero is indicated in gray. Strandness of ncRNAs is included in the biotype names (sense: “_SE,” antisense: “_AS”). Biotypes follow GENCODE/ENSEMBL annotation. Missing annotations are designated as “unknown.”

The selected ncRNA candidates included snoRNAs (11%), miRNAs (14%), as well as—so far—unclassified ncRNA candidates (75%). Thereby, the majority of unclassified RNA candidates mapped to intergenic (50%) or intronic regions of protein-coding genes (40%), genomic locations, which previously have been reported to harbor the majority of known and functional ncRNA species (Fig. 1). Interestingly, 59% of the gene loci, selected for the microarray, could be converted from the mouse (mm9) to the human genome assembly (hg19) by applying the LiftOver tool from the UCSC Genome Browser website. This implies the presence of human homologs of the respective ncRNAs, i.e., evolutionary conservation, and thus is consistent with their biological function.

In addition, we included 259 sequences of computationally predicted ncRNAs in human, which have been converted to the mouse genome, located in introns (83%), exon/intron boundaries (3%), antisense to exons (12%), or close to protein-coding genes (2%), which previously have been reported to be associated with CNS disorders including Alzheimer's Disease (AD), amyotrophic lateral sclerosis (ALS), anxiety, Creutzfeldt–Jakob disease (CJD), multiple-system atrophy (MSA), pain, Parkinson's disease (PD), or progressive supranuclear palsy (PSP), respectively (see Materials and Methods, and Supplemental Fig. S1 for details).

In total, we selected 1472 neuronal or neural-derived ncRNA candidates, including computational ncRNA predictions (see above), as well as experimentally verified candidates, for microarray design. Accordingly, we designed 3977 complementary DNA oligonucleotide probes that covered all 1472 ncRNA candidates of interest (see Materials and Methods). Each oligonucleotide probe set contained one perfect complementary (PM) oligo for short ncRNAs (<70 nt), and multiple PM oligonucleotides for ncRNAs >70 nt. In order to assess for specificity, each PM oligonucleotide was spotted next to a mismatched control oligonucleotide (designated as MM) that harbored a single nucleotide mismatch at position 13 (see Materials and Methods for details).

### Validation of the neuro-ncRNA microarray by expression analysis of ncRNAs between different mouse tissues

To optimize hybridization conditions as well as signal-to-noise ratio, we performed self–self hybridization experiments with RNA from mouse brain, labeled by Cy3 or Cy5, respectively. Since we aimed to focus on small- and medium-sized ncRNA species, hybridization with size selected (i.e., 18–400 nt) RNAs showed a much higher specificity and a significantly higher signal-to-noise ratio compared with hybridization with total RNA and thus was used in following experiments (data not shown; see Materials and Methods). Notably ∼90% of ncRNAs selected from RNA-Seq and ∼50% of ncRNA predictions passed the quality criteria (see Materials and Methods) while no ncRNA candidate was misclassified as differentially expressed (see Supplemental Fig. S2).

**FIGURE 2.**
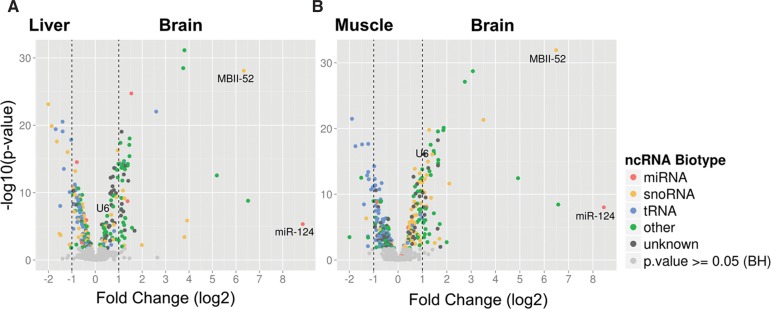
Differential expression analysis of ncRNAs between brain, liver, and muscle tissues, respectively. Volcano plots illustrate ncRNA levels of (*A*) liver and (*B*) muscle compared with brain tissue. ncRNAs with significant changes in expression are indicated by colored dots. Biotypes are designated by color code: miRNAs (red), snoRNAs (orange), tRNA (blue), other (green), and unknown biotype (gray). Vertical dashed lines indicate a twofold change in expression.

As a first proof of principle, we compared ncRNAs expression in brain, muscle, and liver tissues from mouse, respectively, by the microarray. The analysis revealed several novel ncRNAs, which exhibited differential expression between the three tissues; in addition, abundant expression of the neuro-specific control ncRNAs MBII-52 and miR-124 in brain compared with liver and muscle could be verified (Fig. 2A,B). Interestingly, expression of U6 snRNA was up-regulated by twofold in brain compared with muscle tissue, and slightly less up-regulated when compared with liver, consistent with previous observations by Lim et al. (2005).

About one-third of all the ncRNA candidates, present on the microarray, were differentially expressed when comparing brain with muscle or liver tissues. The expression of 20% and 17% of ncRNAs was up-regulated, while the expression of 19% and 17% of ncRNA candidates was down-regulated in brain compared to muscle or liver, respectively (Fig. 2A,B). The apparent bias toward higher expression differences of up-regulated ncRNAs in the brain (Fig. 2A,B) might be explained by the method of ncRNA candidate selection for microarray design, which was based on sequencing data from neural tissues (see Materials and Methods).

**FIGURE 3.**
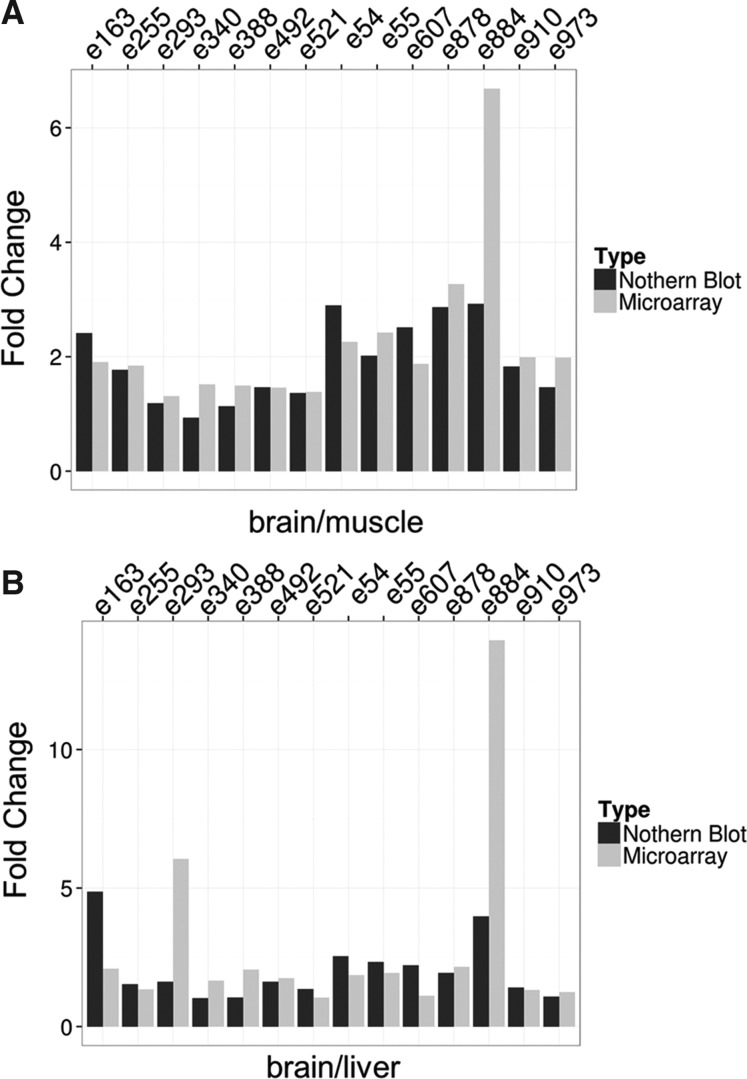
Comparison of expression analysis between Northern blot (black bars) and microarray analysis (gray bars) of 14 randomly chosen ncRNAs. (*A*) Expression changes of ncRNAs between brain and muscle tissues. (*B*) Expression changes of ncRNAs between brain and liver tissues. Candidate IDs are indicated on *top*. Details are listed in Supplemental Table 2. Fold changes from Northern blot analysis were quantified by using ImageQuant 8.1 (GE Healthcare).

We subsequently verified expression changes assessed by microarray analyses with Northern blotting. Thereby, we performed Northern blot analysis of 14 randomly chosen ncRNA candidates, which indicated differential expression in brain compared with liver and muscle according to custom neuro-ncRNA microarray analysis. Indeed, as assessed by Northern blotting, expression changes of all ncRNAs tested correlated well with microarray analysis (Fig. 3A,B). In summary, by these analyses we could demonstrate the suitability of the neuro-ncRNA microarray for expression profiling of ncRNAs in brain tissues of mouse models for CNS diseases.

### Differential expression of ncRNAs in mouse models for psychiatric disorders or Parkinson's disease and Alzheimer's disease

For screening mouse models for CNS diseases for changes in ncRNA expression, we first investigated two models with impaired voltage-gated Ca^2+^ channel activity, i.e., the lethargic mutant of the auxiliary calcium channel β_4_ subunit (Cacnb4^lh^) (Burgess et al. 1997) and knockout mice for the L-type calcium channel Ca_V_1.3 (Platzer et al. 2000), which have been implicated in a variety of neurological disorders such as psychiatric disorders (Cacnb4) or Parkinson's disease (Ca_v_1.3). We followed these investigations by ncRNA expression analysis in a triple-transgenic mouse model mimicking pathophysiological aspects of Alzheimer's disease (AD).

### Differential expressed ncRNAs in lethargic mice exhibiting a Cacnb4 mutation

The first model represented the mouse strain lethargic (lh), which arose spontaneously in inbred BALB/cGn mice, and which harbors a mutation in the gene encoding the voltage-dependent Ca^2+^ channel β_4_ subunit (Cacnb4) leading to a complete loss of the β_4_ protein (Burgess et al. 1997). Mice homozygous for the mutation are first recognized at day 15 by their lethargic behavior with gait instability (ataxia) and occasional seizures, which resemble human petit mal seizures. Because a mutation in the human Cacnb4 gene causes a juvenile form of epilepsy, lethargic mice are frequently considered as a model for idiopathic epilepsy (Escayg et al. 2000).

In addition to its function in calcium channel modulation at the plasma membrane, a second role of the β_4_ protein in gene regulation has recently been suggested (Subramanyam et al. 2009). It has been demonstrated, that splice variants of the β_4_ subunit (i.e., β_4a_ and β_4b_) are able to shuttle to the nucleus and modulate gene expression in neurons, thus playing a dual role in regulating Ca^2+^ channel activity (Etemad et al. 2014). This study postulated a novel feedback mechanism, by which β_4_ splice variants regulate the expression of genes involved in neuronal excitability (including its pore forming partner Ca_V_2.1) in an activity-dependent manner. Considering the high expression of β_4_ in cerebellar neurons this mechanism is likely to be relevant, in particular in the cerebellum.

**FIGURE 4.**
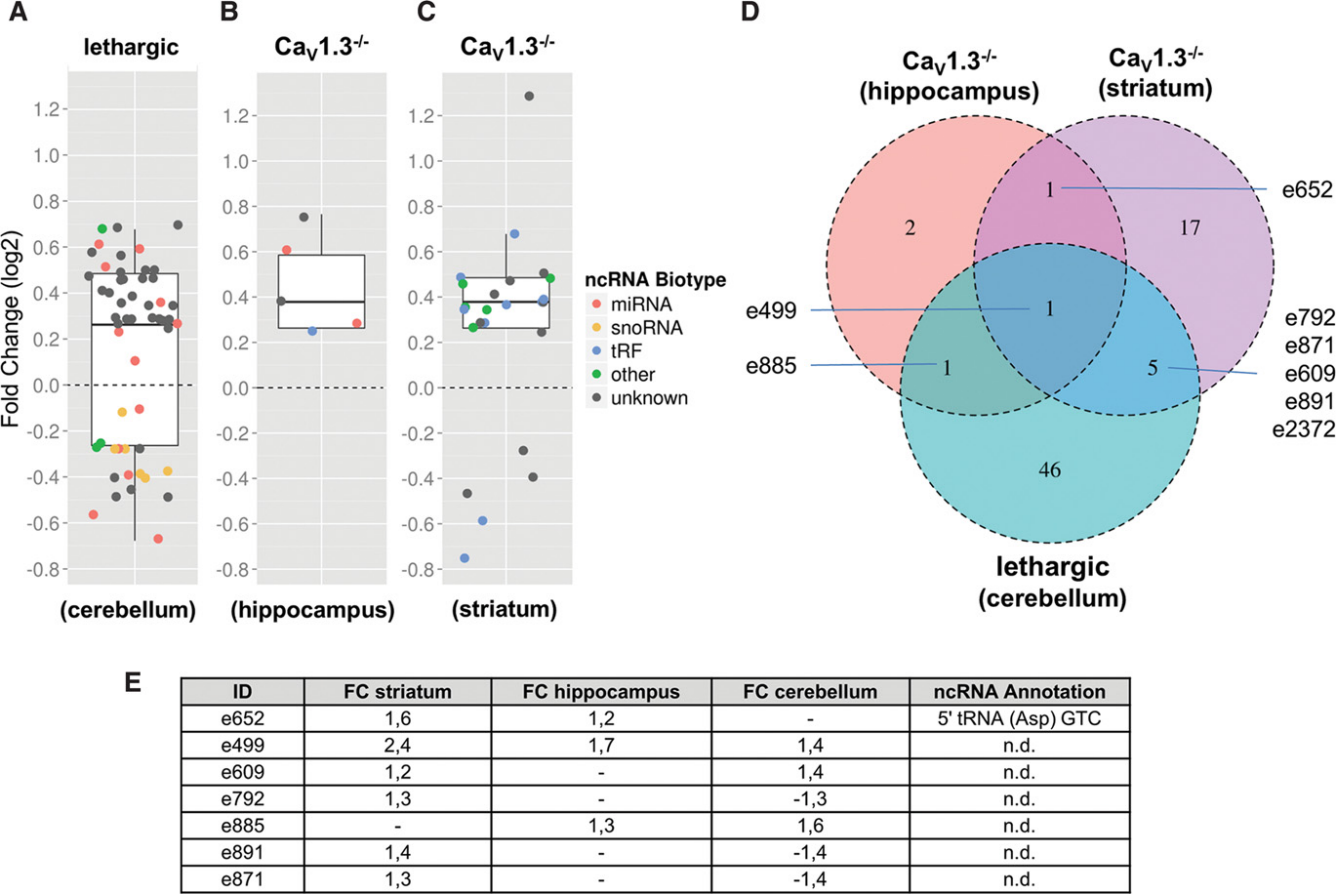
Differential expression analysis of ncRNAs from selected brain regions of mouse models for voltage-gated calcium channel activity compared with wild type. (*A*) Cacnb4 mutant mouse: cerebellum. (*B*) Ca_V_1.3^−/−^: hippocampus. (*C*) Ca_V_1.3^−/−^: striatum. Box plots represent fold changes of ncRNAs (colored data points), which showed significant changes in expression relative to wild-type controls in microarray experiments. Fold changes are illustrated in log_2_ scale. Whiskers extend to ±1.5-fold of the interquartile range (IQR). Biotypes are designated by color code: miRNAs (red), snoRNA (orange), tRF (blue), other biotypes (green), and unknown biotype (gray). (*D*) Venn diagram illustrating the overlap of differentially expressed candidates between the models analyzed. (*E*) Fold change and annotation of ncRNA candidates highlighted in *D*.

In order to analyze whether the presumed gene-regulatory function of the β_4_ subunit would also extend to ncRNA expression, we performed a neuro-ncRNA microarray analysis from cerebella of 2 mo homozygous lethargic (*lh*/*lh*) mice compared with wild-type littermates. Indeed, this analysis revealed the differential expression of 53 ncRNA candidates (Fig. 4A). While 20 of these belonged to known classes of ncRNAs (i.e., miRNAs, snoRNAs, or snRNAs, respectively), 33 represented so-far unclassified ncRNAs.

Interestingly, 10 of the differentially expressed miRNAs are predicted to target at least one mRNA related to calcium channel function, based on the miRNAmap resource (http://mirnamap.mbc.nctu.edu.tw/) and therefore might be involved in Ca^2+^ homeostasis (Supplemental Table 1). In particular, mmu-miR-221-3p (e2246) (Supplemental Table 1) and mmu-miR-222-3p (e2248) (Supplemental Table 1), whose expression were both down-regulated in the absence of the β_4_ subunit, are predicted to directly target the Cacnb4 mRNA (Supplemental Table 1), thereby possibly indicating a feedback mechanism. In general, these data are consistent with a previously suggested feedback loop, which links presynaptic calcium channel activity with transcriptional regulation (Etemad et al. 2014).

In addition, loss of the β_4_ subunit might also affect the glutamate receptor signaling (NMDA) cascade in the cerebellum by the observed up-regulation of mir-219a-5p (e2150) (Supplemental Table 1). This miRNA has previously been shown to directly target the calcium/calmodulin-dependent protein kinase II γ subunit (CaMKIIγ), an essential component of the NMDA receptor signaling cascade (Kocerha et al. 2009). NMDA receptor signaling is essential for the communication of climbing fibers to Purkinje cells (Piochon et al. 2010) and thereby regulates cerebellar plasticity, which is significantly disturbed in lethargic mice.

Thus, a significant number of the predicted as well as validated mRNA targets of the differentially expressed miRNAs identified can be associated with calcium signaling and therefore might also support the biological relevance of the remaining, entirely novel ncRNAs. In the future, it will be important to address the biological roles and targets of these novel ncRNAs, which might be either involved in calcium homeostasis or signaling, as observed for miRNAs. Considering that the β_4_ subunit plays a dual role in calcium channel modulation (see above), differential expression of ncRNAs could be caused either indirectly by a mechanism involving calcium channel function, or be directly related to the regulatory role of the β_4_ subunit in the nucleus in transcription.

### Changes of ncRNA expression in Ca_V_ 1.3 knockout mice

Since Ca_V_1.3 is critically involved in several general mechanisms regulating neuronal plasticity and synapse morphology, we next investigated ncRNA expression in Ca_V_1.3 knockout (Ca_V_1.3^−/−^) mice (Platzer et al. 2000). The Ca_V_1.3 subunit is predominantly expressed in neurons, neuroendocrine cells, sensory cells, and cardiac pacemaker cells. Ca_V_1.3^−/−^ mice are deaf due to the absence of Ca_V_1.3 LTCC currents in cochlear inner ear hair cells, and suffer from bradycardia. These channels play also a delicate role in synaptic plasticity with knockout animals showing reduced drug-taking behaviors and an antidepressant-like phenotype (for review, see Striessnig and Koschak 2008; Striessnig et al. 2014).

Analysis of hippocampi and striata of Ca_V_1.3^−/−^ mice compared with wild-type control animals revealed 5 and 24 differentially expressed ncRNAs, respectively. From the 29 ncRNAs, 14 can be assigned to known classes of ncRNAs (i.e., two miRNA, 7SK RNA, Y RNA, and 10 tRNA-derived RNAs) while the remaining 15 ncRNAs represented currently unclassified ncRNA species (Fig. 4B,C). Expression of two miRNAs, designated as mmu-miR-204-5p (e3080) (Supplemental Table 1) and mmu-miR-143-3p (e2138) (Supplemental Table 1), was up-regulated in hippocampi of knockout mice compared with wild-type controls. Interestingly, both miRNAs are predicted to target the 3′ UTRs of several ion channel mRNAs indicating potential cross-regulatory effects (see Supplemental Table 1). Consistent with this model, mmu-miR-204-5p is located in an intron of the transient receptor potential cation channel, Trpm3.

In total, expression of eight ncRNA candidates was deregulated in at least two brain regions between the two calcium channel mouse models (Fig. 4D,E). In particular, we identified one ncRNA candidate (designated as e499) (Fig. 4D; Supplemental Table 2), which was found to be differentially expressed in both Ca^2+^ channel mouse models and also in all brain tissues tested (Fig. 4D). The expression of this ncRNA candidate is up-regulated by 1.7-fold in hippocampi, 2.4-fold in striata of the Ca_V_1.3^−/−^ mice, and 1.4-fold in cerebella of lethargic (Cacnb4) mice. The ncRNA candidate is located in the fifth intron of the vomeronasal 2, receptor 63 (Vmn2r63) a reported pseudogene, which belongs to the vomeronasal 2 receptor family that is involved in sensing of pheromones.

### ncRNAs expression profiling of Alzheimer's disease (AD) mouse model

Alzheimer's disease (AD) is a severe chronic brain disease and the most common form of dementia in the western world (Soldano and Hassan 2014). Extracellular depositions of β-amyloid aggregates (plaques) and intraneuronal inclusions of Tau protein (i.e., neurofibrillary tangles) are the major pathophysiological hallmarks of AD (Cummings et al. 1998). In addition, neuro-inflammation, cerebrovascular dysfunction including blood–brain barrier breakdown, cell death of cholinergic neurons, activation of microglia and astroglia and oxidative stress have been reported (Iadecola 2013). While only <5% of AD cases exhibit a genetic predisposition, the majority (>95%) is sporadic with age as the main risk factor. In order to study AD, several transgenic animal models have been developed to mimic its pathophysiological hallmarks (Gotz and Ittner 2008; Hall and Roberson 2012). In this study, we have investigated a well-established triple-transgenic AD model, described by Oddo et al. (2003). These mice express presenilin PS1_M146V_, APP_Swe_, and tau_P301L_ transgenes, respectively, and develop β-amyloid plaques and, at later stages, also a tau pathology (Oddo et al. 2003).

We applied the novel neuro-ncRNA microarray platform to differential expression analysis of ncRNAs from prefrontal cortices of 3-, 10-, and 20-mo-old triple-transgenic (3xTG) mice of the Alzheimer's disease models versus control animals. Three and 10-mo-old mice have previously been reported (Hunter et al. 2011) to lack β-amyloid plaques, as well as tau tangles, whereas the 20-mo-old animals showed substantial plaque formation in the cortex (Hunter et al. 2011). First behavioral defects have been detected in the age group of 10 mo (data not shown). The 3-mo-old mice are largely without any obvious AD-related phenotype (C Humpel, pers. comm.).

### Differentially expressed ncRNAs in young mice of the 3xTG model for AD

We first investigated expression changes of ncRNA candidates before the occurrence of extracellular β-amyloid plaques, as well as tau tangles, since we hypothesized that expression of ncRNAs—regulating protein-coding genes—might precede the appearance of AD specific symptoms. Thereby, these “early-onset” ncRNAs might also serve as potential candidates for early diagnosis of AD in the future. This hypothesis is supported by the observation of differentially expressed miRNAs, such as miR-107, by Wang et al. (2008) in young transgenic mouse models, showing no phenotypical hallmarks of AD.

**FIGURE 5.**
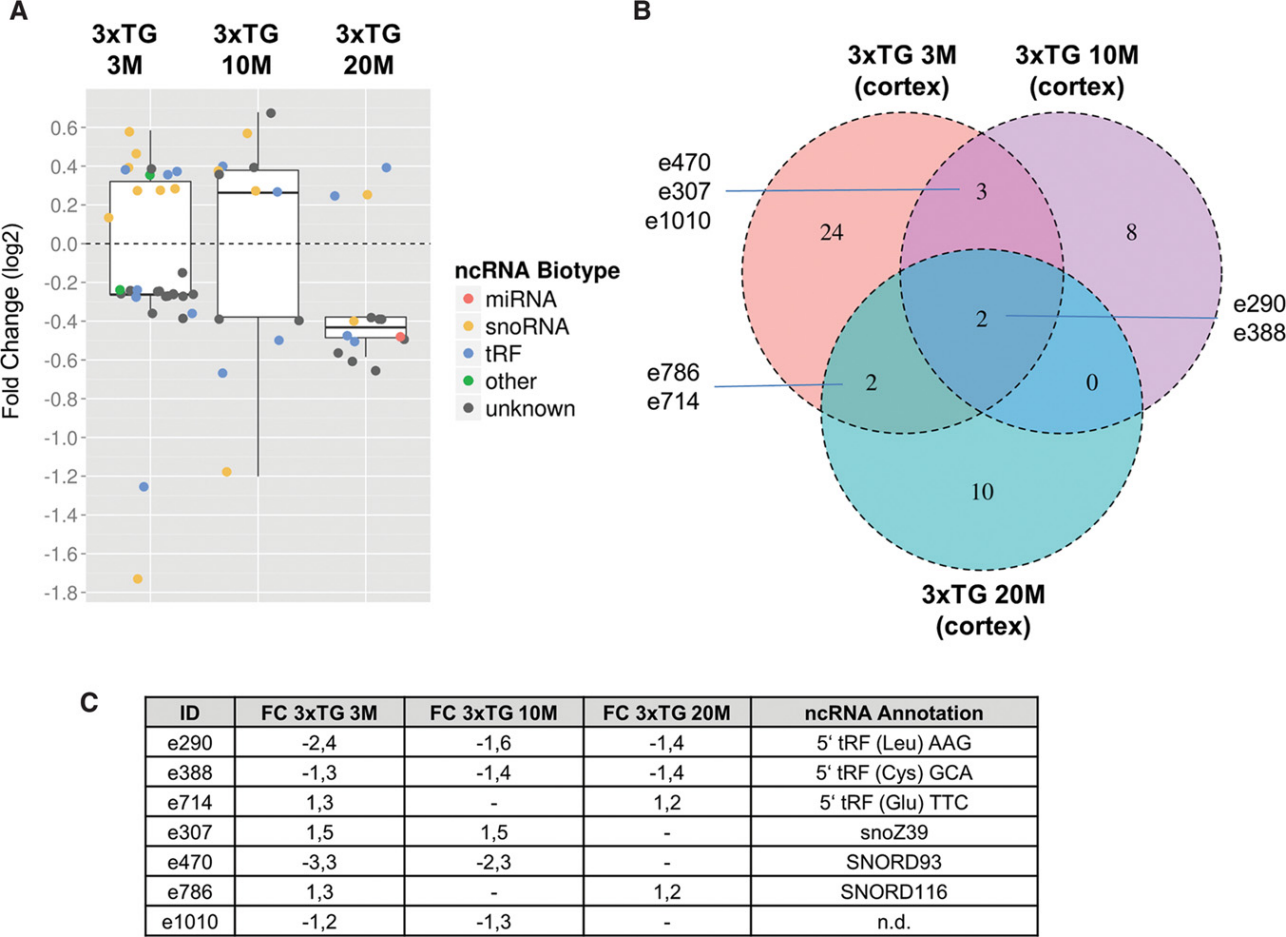
Differential expression of ncRNAs from cortices of a triple-transgenic (3xTG) mouse model for Alzheimer's disease (Oddo et al. 2003) compared with wild-type controls at the age of 3, 10, and 20 mo, respectively. (*A*) Box plots represent fold changes of ncRNAs (colored data points), which showed significant changes in expression relative to their wild-type controls in microarray experiments. The experiments are illustrated from *left* to *right*: 3-, 10-, and 20-mo-old mice. Fold changes are illustrated in log_2_ scale. Whiskers extend to ±1.5-fold of the interquartile range (IQR). Biotypes are designated by color code: miRNAs (red), snoRNA (orange), tRF (blue), other biotypes (green), and unknown biotype (gray). (*B*) Venn diagram illustrating the overlap of differentially expressed candidates between the different age groups. (*C*) Fold change and annotation of ncRNA candidates highlighted in *B*.

Hence, we performed ncRNA expression profiling of cortices from 3-mo-old 3xTG mice compared with wild-type controls by the neuro-ncRNA microarray, which revealed 31 ncRNAs, whose expression was up- or down-regulated, respectively. From these, we identified eight snoRNAs, seven tRNA-derived RNAs, and 16 currently unclassified ncRNA candidates (Fig. 5A). To investigate expression changes of ncRNAs during aging just before β-amyloid plaque formation we performed expression profiling of cortices from 10-mo-old 3xTG mice compared with wild-type controls. This analysis revealed 13 differentially expressed ncRNAs. From these, four ncRNAs were identified as snoRNAs, four are derived from tRNAs, while an additional five ncRNAs represented currently unclassified RNA species (Fig. 5B).

**FIGURE 6.**
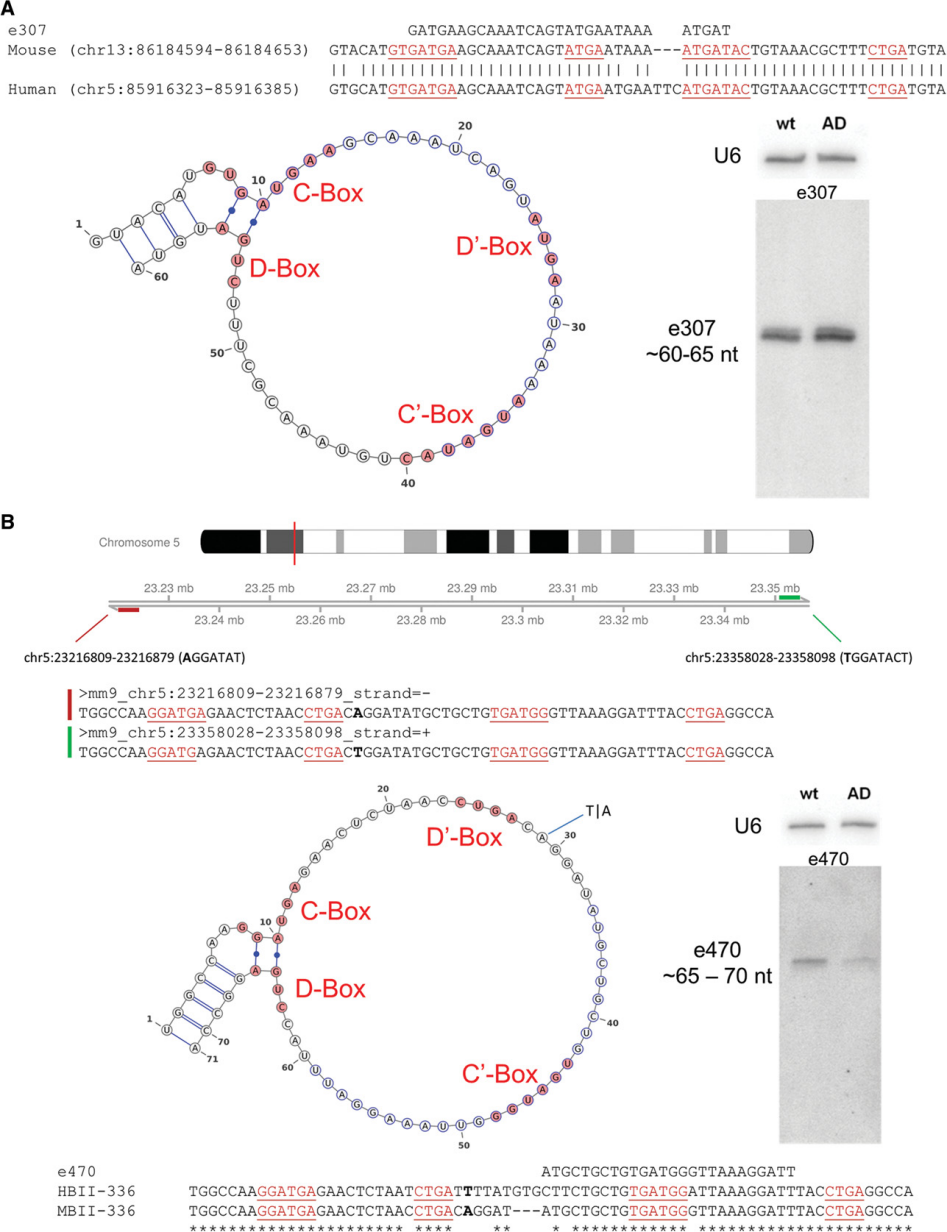
Differentially expressed snoRNAs in young 3xTG AD mice. (*A*) Candidate e307: sequence similarity of mouse (mm9 assembly) to the human (hg19 assembly) snoRNA homologs (located on chromosome 5), and (*left*) predicted secondary structure illustrated by VARNA (http://varna.lri.fr/). (*Right*) Northern blot analysis of e307 from total RNA of cortices of 10-mo-old 3xTG mice compared with wild-type controls. (*B*) Candidate e470: genome positions of the two (red and green bars) potential loci of transcription (on chromosome 5), which differ by a single SNP (indicated in bold). Sequence similarity of HBII-336 and MBII-336; (*left*) predicted secondary structure illustrated by VARNA. (*Right*) Northern blot analysis from total RNA of cortices of 10-mo-old 3xTG mice compared with wild-type controls. C/D boxes are illustrated in red and sequences targeted by the oligonucleotide from the microarray e307 and e470, respectively, are highlighted in blue.

Among the most significantly differentially expressed ncRNAs we identified two predicted C/D box snoRNAs (e307 and e470) (Fig. 6A,B; Supplemental Table 2), based on conserved sequence and structure motifs (Bachellerie et al. 2002), termed as e307 and e470, respectively. Expression of e307 and e470 was deregulated in both, the 3- and 10-mo-old 3xTG mice (Fig. 5A–C). Thereby, expression of e307 was up-regulated in both age groups, whereas expression of e470 was down-regulated in the 3- and 10-mo-old 3xTG mice, respectively (Fig. 5B,C). Thus, expression deregulation of these snoRNAs occurs prior to β-amyloid plaque formation. Differential expression of e307 and e470, respectively, was subsequently also confirmed by Northern blot analysis (Fig. 6A,B) and qPCR (data not shown).

According to bioinformatical analysis, e307 represents a not yet reported C/D box snoRNA in mouse but is highly similar (92% sequence identity) to human snoRNA ZL107 (Fig. 6A; Kishore et al. 2013) and contains, within its sequence, the predicted human miRNA hsa-mir-3607. e470 represents a previously reported C/D box snoRNA in mouse, designated as SNORD93 (Huttenhofer et al. 2001). Thereby, the sizes of the ncRNAs are smaller than the average length of canonical snoRNAs, i.e., ∼65 nt according to Northern blot analysis (Fig. 6A,B). Although previously, e307 has been reported to encode human miRNA-3607 (see above), we were unable to detect any signals for an RNA species of that size by Northern blotting (Fig. 6A).

Hence, we propose that both ncRNAs represent bona fide snoRNAs since they contain conserved C/D box sequence elements, i.e., a C box (RUGAUGA, R = G or A) and a D box (CUGA) of canonical C/D box snoRNAs (Fig. 6A,B), a class of small ncRNAs guiding 2′-*O*-methylation of rRNA or snRNA molecules (Cavaille et al. 2000; Kiss 2002). As reported for canonical snoRNAs, e307 is localized within the intron (second) of a protein-coding gene, Cox7c (cytochrome c oxidase subunit 7C). Cox7c is described in the Alzheimer's disease pathway (ko05012) according to the Kyoto Encyclopedia of Genes and Genomes (KEGG) (Kanehisa 2002). Thereby, it seems to be involved in mitochondrial dysfunction (Weydt et al. 2006), which is reported to be a prominent feature of AD (Wang et al. 2014). e470 maps to the second intron of lncRNA AI506816. However, according to RNA-seq data, a single nucleotide polymorphism (SNP) at position 29 in the snoRNA (Fig. 6B) reveals a second location in the second intron of the putative long intergenic ncRNA (lincRNA) 2700038G22Rik (Mouse Genome Informatics MGI).

As reported for canonical snoRNAs, both snoRNAs e307 and e470 are predicted to guide 2′-O-ribose-methylation of 18S ribosomal RNA (rRNA), at positions C_798_ and U_576_, respectively. In line with these predictions, both positions are reported to be methylated in human 18S rRNA. Differential expression of both snoRNA candidates is no longer observed upon plaque formation in 20-mo-old 3xTG mice (see below), which might be caused by plaque deposition potentially suggesting an early pathophysiological role of e307 and e470 prior to plaque formation (Oddo et al. 2003). Although at this point the biological role of these snoRNAs in the etiology of AD remains currently elusive, they could be used as early diagnostic markers in AD in the future (see below).

### Differentially expressed ncRNAs in old mice of the 3xTG model for AD

Expression profiling of cortices from 20-mo-old 3xTG mice compared with wild-type controls revealed differential expression of 14 ncRNAs. From these, seven can be assigned to known classes of ncRNAs (i.e., two snoRNAs, one miRNA, and four tRNA-derived sequences) and seven represented currently unclassified ncRNAs (Fig. 5A).

From ncRNAs of known RNA classes, expression of miRNA mmu-miR-195-5p (e2030, Supplemental Table 2) is down-regulated in the 20-mo-old 3xTG mice. mmu-miR-195 has previously been reported to target the mRNA of the beta-site APP cleaving enzyme 1 (BACE1) in SAMP8 mice, which down-regulates β-amyloid production by inhibiting expression of BACE1 (Zhu et al. 2012). This negative feedback mechanism may also be present in this mouse model, since mice show the presence of amyloid plaques at 20 mo of age.

In addition, SNORD116 (e786, Supplemental Table 2), a C/D box snoRNA previously reported to be involved in the etiology of the Prader–Willi Syndrome (PWS) (Cavaille et al. 2000; Skryabin et al. 2007; Sahoo et al. 2008), was also found to be down-regulated in the 20-mo-old 3xTG mice (Fig. 5B,C). PWS is a neurodevelopmental disease, generally caused by a 4 Mb deletion on chromosome 15q11-13, a region which has been reported to be paternally imprinted (Runte et al. 2001; Stefan et al. 2005). In addition to severe obesity due to hyperphagia, PWS patients also exhibit behavioral deficits such as varying grades of mental retardation (Roof et al. 2000). It will be interesting to investigate potential overlaps in the etiology of these two neurological diseases due to differential expression of SNORD116.

In the 3-, 10-, and 20-mo-old mice of the AD model we identified seven ncRNAs whose expression was found to be deregulated in at least two time points (Fig. 5B,C). From these, expression of two ncRNAs was down-regulated at all three time points. Interestingly, both ncRNA, i.e., e388 and e290, represent 5′-derived fragments of tRNAs (i.e., from tRNA^Cys^ and tRNA^Leu^ respectively), as assessed by RNA-Seq data from ES cell library analysis from which sequences were taken for selection of ncRNA candidates for the microarray (Supplemental Fig. S3C,D).

Indeed, Northern blot analysis of total brain confirmed the presence of tRNA-derived fragments for both candidates (see Supplemental Fig. S3A,B). Thereby, tRNA fragments, detected by Northern blot analysis are sized between 30 and 35 nt and might therefore represent so-called tRFs (tRNA-derived-fragments), which recently have been described to be stress-induced in mammalian cells and have been suggested to inhibit translation by targeting the translational initiation machinery (Emara et al. 2010).

Thereby, tRFs can derive either from 5′- or 3′-ends of full length tRNAs, respectively, designated as 5′-tRFs or 3′-tRFs. It has been proposed that tRFs might be generated by miRNA related processing enzymes such as Dicer (Babiarz et al. 2008) and thus might fulfill similar functions in regulation of gene expression. Although neuro-ncRNA microarray analysis does not allow to distinguish between full length tRNAs or 5′ and/or 3′ tRNA fragments, we were able to demonstrate the presence of defined tRNA fragments for these two tRNA species in total brain RNA of wild-type animals (Supplemental Fig. S3A,B).

## CONCLUSION

In contrast to commercially available miRNA microarrays, aiming for expression analysis of unstructured small ncRNAs of identical size, the generation of a custom-made neuro-ncRNA microarray, encompassing novel and known ncRNA species possesses several challenges: (a) Due to secondary/tertiary structures of ncRNAs and their heterogeneous length, hybridization of fluorescently labeled ncRNAs to oligonucleotide probes requires careful selection of probes; and (b) cross-hybridization of oligonucleotide probes, resulting in false positive hybridization signals, had also to be taken into account. In this study, by generation of an algorithm for proper selection of oligonucleotide probes and also by including mismatched oligonucleotide controls, we were able to successfully generate the first highly accurate, specific, and sensitive neuro-ncRNA microarray for analysis of differential expression of ncRNAs involved in CNS diseases. Unlike for RNA-seq analysis, the neuro-ncRNA microarray enables a fast and efficient expression analysis of a large number of samples with little computational analysis needed.

First, as a proof of principle, we compared ncRNA expression between brain, liver, and muscle tissues from mouse and thereby identified well over a hundred novel ncRNA species, with higher expression in brain tissue compared with muscle or liver and ∼100 ncRNAs with lower expression in brain. The remaining novel ncRNA candidates, spotted on the microarray, were similarly expressed in all three tissues indicating that exclusive brain-specific expression of small ncRNAs is rare.

We applied the neuro-ncRNA microarray to two mouse models with impaired voltage-gated calcium channel activity, i.e., to Cacnb4 mutant lethargic and Ca_V_1.3 knockout mouse, implicated in various neurological disorders such as psychiatric disorders or Parkinson's disease. These mouse models revealed 12 differentially expressed miRNAs targeting the 3′ UTRs of genes involved in calcium signaling. These findings thus might be consistent with a biological relevance of 40 entirely novel ncRNAs, identified in this study. In particular, we identified one ncRNA candidate (designated as e499), which was found to be differentially expressed in both mouse models and also in all brain tissues investigated. Such a finding is not unexpected because b4 subunits (encoded by the Cacnb4 gene) are also associated with LTCCs such as Cav1.3 in the brain (Pichler et al. 1997). The ncRNA candidate e499 is located in the fifth intron of the vomeronasal 2, receptor 63 (Vmn2r63) a pseudogene of the vomeronasal receptor 2 family, involved in sensing of chemical stimuli in rodents (Ryba and Tirindelli 1997); interestingly, it has been reported that in Parkinson's disease in ∼75% of cases scenting ability is reduced (Wenning et al. 1995).

Analysis of the AD mouse model revealed two snoRNAs, designated as e307 and e470, respectively, which showed differential expression in young AD mice (i.e., 3 and 10 mo old) compared with wild-type controls. Interestingly, by qPCR we were able to detect both snoRNAs within human CSF samples of healthy individuals (data not shown); thus, these snoRNAs might be used as biomarkers for early diagnosis of Alzheimer's disease in humans. In general, ncRNAs might represent ideal biomarkers, compared with proteins and/or mRNAs, because (a) of an unprecedented sensitivity of detection by qPCR (i.e., up to single molecules) and (b) because of their enhanced stability, due to secondary and tertiary structures and by binding to proteins, thereby forming ribonucleoprotein complexes (RNPs).

Although highly significant and validated by Northern blotting, expression differences of ncRNAs in the calcium channel, as well as in the AD mouse models, compared with wild-type controls were found to be moderate. Although this could reflect an intrinsic limitation of our customized microarray, we obtained identical results for the subclass of identified, differentially expressed miRNAs by using commercially available microarrays (i.e., Exiqon) or by Northern blot and qPCR analysis, respectively. Hence, two explanations for observed moderate expression changes can be envisioned: (1) Since entire brain areas were used in expression analyses, such as hippocampus, striatum, cerebellum, or cortex, differential expression of ncRNAs might occur in certain cell types only, and thus might be masked by a high background of unchanged expression in all other cell types. (2) The brain, as one of the most sophisticated organs within the human body, might demand precise fine-tuning of gene expression, a task generally exerted by ncRNAs, and hence observed mild changes in expression of ncRNA changes would reflect this requirement, which would be in agreement with earlier findings by Lau et al. (2013).

In conclusion, generation of a neuro-specific ncRNA microarray in this study enabled the identification of a large number of ncRNA candidates potentially involved in CNS diseases. Notably, we found a number of differentially expressed noncoding RNAs in an ongoing project, by focusing on the peripheral nervous system. Therefore, the microarray could also be used for the analysis of other tissues and diseases, including human tissue samples, since 66% of the ncRNA candidates present on the neuro-ncRNA microarray could also be mapped to the human genome. In the future, it will be interesting to study the pathophysiological and biological functions of all newly discovered RNA molecules in the brain.

## MATERIALS AND METHODS

### Development of custom neuro-ncRNA microarray: selection of ncRNA candidates

The custom neuro-ncRNA microarray for expression analysis of newly identified small ncRNAs (18–400 nt) from murine neural tissue was designed based on (1) RNA sequencing (RNA-Seq) data and (2) computational prediction of small ncRNAs.

All ncRNA candidates were selected from RNA-Seq data obtained from (a) murine ES cells differentiated into neural cells, resulting in three successive stages of differentiation (Skreka et al. 2012), (b) whole mouse brain (Rederstorff et al. 2010), and (c) dorsal root ganglia from embryos as well as adult mice (K Skreka, C Bandtlow, G Dechant, M Rederstorff, and A Hüttenhofer, in prep.). RNA-Seq data were mapped to the mouse genome (mm9) and annotated by APART, a specialized pipeline for the analysis of short RNA sequencing data and annotation of short ncRNAs, developed in our laboratory (Zywicki et al. 2012). An ncRNA locus required a representation by a minimum of five reads from deep sequencing in order to be considered for microarray design (Rederstorff et al. 2010; Skreka et al. 2012). Furthermore, selection of ncRNA candidates was based on observed changes in expression during differentiation of ES cells into neural cells (Skreka et al. 2012). From these data, we obtained 1213 assembled contiguous sequencing reads (contigs) for oligonucleotide design, including snoRNAs (11%), tRNA-derived sequences (6%), miRNAs (13%), and unclassified ncRNA candidates (70%). For contigs <70 nt, one single region (sized 18–30 nt) was selected for subsequent oligonucleotide design, whereas in the case of longer contigs multiple nonoverlapping oligonucleotide regions were selected at intervals of 30 nt. Thereby, we preferably selected regions with elevated read coverage compared with the surrounding sequences.

High read coverage within a predicted ncRNA locus flanked by significantly lower coverage of reads at one or two ends of the contig might be indicative of a potential processing product of a precursor transcript (Zywicki et al. 2012). One low coverage region per contig (read coverage below one-third of the highest coverage) was selected representing control/precursor regions.

Computational ncRNA predictions, included for microarray design, were taken from the study by Gorodkin et al. (2010). Only those ncRNA gene predictions were included which were located in introns (83%), exon/intron boundaries (3%), and antisense to exons (12%), or close to protein-coding genes (2%), which were reported to be associated with CNS disorders such as Alzheimer's disease (AD), amyotrophic lateral sclerosis (ALS), anxiety, Creutzfeldt–Jakob disease (CJD), multiple-system atrophy (MSA), pain, Parkinson's disease (PD), or progressive supranuclear palsy (PSP) from MalaCards (Rappaport et al. 2013) and BioGraph (Liekens et al. 2011).

In addition, ncRNA predictions from intergenic regions between protein-coding genes (2%) in close proximity (within 500 nt) to a SNP associated with CNS disorders were considered for microarray design. Subsequently, the human ncRNA predictions of interest were mapped to the mouse genome by using the liftOver tool from the UCSC Genome Browser, applying the default parameters (Kent et al. 2002), resulting in 259 ncRNA predictions that were taken for oligonucleotide design as described below. Annotation of ncRNA predictions can be found in Supplemental Figure S1.

### Oligonucleotide design

Selected sequences, as described above, were processed by OligoWiz (Wernersson and Nielsen 2005) to design a representative and compatible oligonucleotide set for hybridization, with (i) a favored melting temperature of 60°C (DNA:RNA temperature model), (ii) an intended oligo length of 25 nt (minimum 18 nt and maximum 30 nt), and (iii) a preference for a position in the center of the selected sequence of interest. The emphasis on the parameters for oligo design was set as follows: 24.2% cross-hybridization, 32.3% delta TM, 6.1% folding energy, 11.3% position within the sequence, and 16.1% on the low-complexity score. Finally, a set of eight random spike-in probes and 40 probes for expression analysis of U2 and U6 snRNAs as internal controls were included on the microarray, resulting in a total of 2081 oligonucleotides. Oligonucleotides designed based on RNA-Seq experiments and ncRNA predictions were spotted in pairs, consisting of a perfect complementary (PM) and a mismatch (MM) oligonucleotide, comprising one mismatch at position 13. Notably G–T juxtaposition was not permitted in the MM probe design. This allows for assessing the specificity of the microarray results and also facilitates data filtering (see Gene Expression Analysis). The oligonucleotides selected as control regions (see above) were spotted without the MM oligonucleotide. Altogether we spotted 3977 oligonucleotides onto the microarray that covers all 1472 ncRNA candidates, including snoRNAs (9%), miRNAs (11%), unclassified ncRNA candidates (62%), and ncRNA predictions (18%).

### Microarray generation

The neuro-ncRNA microarray was generated using the MicroGrid II Microarray Spotter (Zinsser Analytic). Thereby, 3977 oligonucleotides were dissolved in spotting buffer consisting of 3× SSC, 1.5 M betaine, yielding a concentration of 25 µm, and spotted on HiSens epoxy-coated glass slides (SCHOTT). All 3977 oligonucleotides were spotted twice in quadruplicates—yielding octuplicates—in a spatial distribution to minimize negative effects of local hybridization artifacts. The oligonucleotides were then covalently bound to the epoxy-coated glass slides with their amino-modified 5′-ends by baking for 1 h at 120°C.

### RNA isolation and RNA size fractionation

Fresh tissue samples from mouse brain regions were snap frozen in liquid nitrogen and stored at −80°C up to 6 mo. Total RNA was isolated by Tri-Reagent (Sigma) according to the manufacturer's instructions and dissolved in DEPC-water. Total RNA was size-fractionated on denaturing polyacrylamide gels (PAGE). To that end, 10–50 µg of total RNA was heat-denatured for 3 min at 95°C in formamide buffer and separated on an 8% denaturing polyacrylamide gel. RNA was visualized by ethidium bromide, and RNA molecules between 18 and 400 nt were excised and subsequently eluted in 300 mM NaCl, 0.2% SDS, and 60 mM NaOAc (pH 5.2) overnight at 4°C. RNAs were extracted with phenol–chloroform (PCI), precipitated with isopropyl alcohol, washed with 70% ethanol, and dissolved in DEPC-water.

### RNA labeling

Three hundred seventy-five nanograms of size selected RNA per sample was fluorescently labeled with either Cy3 or Cy5 dyes using the miRCURY LNA microRNA Array Hi-Power labeling kit from Exiqon according to the manufacturer's protocol.

### Microarray hybridization and scanning

Hybridization was conducted in dye-swap experiments and was performed using the Tecan HS400 Pro device. Microarray slides were equilibrated at 38°C with Pre-Hyb-I (5× SSC, 0.1% SDS, 1% BSA) and subsequently with hybridization buffer (5× SSC, 0.1% SDS, 10% formamide, 250 ng/µL total *Escherichia coli* tRNAs), prior to loading of samples to the hybridization chamber. To facilitate hybridization in particular of structured ncRNAs, a temperature gradient was applied. Hybridization was started at 65°C and subsequently reduced to a final temperature of 36°C in 2°C–3°C decrements within a period of 14–16 h. Five consecutive washing steps were performed at 36°C: two with Wash-Buffer I (2× SSC, 0.1% SDS), two with Wash-Buffer II (0.2× SSC, 0.1% SDS), and one with Wash-Buffer III (0.2× SSC). Upon washing, slides were dried by nitrogen gas at 30°C for 5 min. Scanning was performed immediately following hybridization at a resolution of 5 µm using the Tecan Powerscanner device (Tecan Group Ltd.). Microarray scans were quantified using the data analysis software ArrayPro 6.3.

### Experimental design

All experiments were performed in dye swap pairs with three biological replicates from transgenic/mutant mice and age matched control mice, respectively.

### Gene expression analysis

Gene expression analysis was performed, by using the R (R Development Core Team 2013) Bioconductor platform (Gentleman et al. 2004), in particular the limma package (Smyth 2005). Normalization was carried out (a) by calculating a local linear regression based on the print tip groups (Yang et al. 2002) within arrays and (b) by quantile normalization (Yang and Thorne 2003) between arrays. Normalization was based on the net intensity values (raw intensity – local background). Subsequently, normalization was inspected by quality metrics (Kauffmann et al. 2009). Oligonucleotides considered for differential expression analysis were filtered by the criterion that the signal intensity of the perfect complementary (PM) had to exceed that of the mismatch (MM) oligonucleotide under at least one experimental condition (mutant or wild type). Differential expression analysis was performed by calculating a linear model for each ncRNA candidate by following the guidelines for simple dye-swap experiments (Smyth 2005). Duplicated spots were considered in the linear model fit. This model was then used to obtain test statistics by the empirical Bayes method providing stable estimations for the sample variance of a small number of arrays (Smyth 2004). All differentially expressed genes with an adjusted *P*-value <0.05 after multiple testing correction as proposed by Benjamini and Hochberg (1995) were considered statistically significant.

### Northern blot analysis

Northern blots were performed as previously described (Rederstorff et al. 2010). Thereby, DNA oligonucleotide probe sequences were identical to oligonucleotide sequences on the microarray.

### Keeping of mice and tissue preparation

Triple-transgenic animals (strain 004807; B6:129-Psen1tm1Mpm Tg [APPSwe, tau301L] 1Lfa/Mmj) were purchased from The Jackson Laboratory (Maine, USA), in agreement with a condition of supply by the University of California (Frank LaFerla, UCI, USA). Age matched B6129SF2/J mice (strain 101045, Jackson Laboratory) were used as controls. Cav1.3-deficient mice (Cav1.3^−/−^) (Platzer et al. 2000) were back-crossed at least five times in C57Bl/6N background. All mice were kept according to standard animal care protocols and to the national animal welfare bodies, fed ad libitum with regular animal diet and maintained in a pathogen-free environment in single ventilated cages. All experiments were performed using male mice. The transgenic status of each animal was confirmed by real-time PCR of tail snips using specific primers and the appropriate hybridization probe. Animals were anesthetized by subcutaneous sodium thiopental (12.5 mg/mL, 1 mL) injection prior to collecting brain tissue. The brains were removed and the parietal cortices were dissected from the left hemisphere and snap frozen in liquid nitrogen and stored at −80°C for later RNA isolation up to 6 mo. All animal experiments were approved by the Austrian Ministry of Science and Research and conformed to the Austrian guidelines on animal welfare and experimentation.

Lethargic mice (Cacnb4^lh^; 129/SvJ background) were bred and genotyped as previously described (Burgess et al. 1997; Etemad et al. 2014). Tissue was prepared from 2-mo-old male lethargic (Cacnb4^lh/lh^) and littermate wild-type control mice as previously described (Schlick et al. 2010). Briefly, mice were euthanized by CO_2_ exposure and decapitated. Subsequently the skull was opened from caudal to rostral and the brain was carefully removed and placed in ice-cold Hank's buffered salt solution. The entire cerebellum was cut from the brainstem, snap frozen in liquid nitrogen and stored at −80°C for later RNA isolation.

### Genotyping (3xTG AD mice)

All animals have been genotyped according to standardized methods. In short, DNA of 0.5 cm mouse tail snip was extracted using Qiagen's DNeasy Kit according to the manufacturer's instructions. Microsynth established the required primers to detect the APP gene: forward primer 5′-AGGACTGACCACTCGACCAG-3′ and the reverse primer 5′-CGGGGGTGTAGTTCTGCAT-3′. Primers were amplified via polymerase chain reaction (PCR) and the thermocycling conditions were set as follows: initial denaturation at 94°C for 3 min (1 cycle), denaturation at 94°C for 30 sec (35 cycles), annealing at 52°C for 1 min (35 cycles), extension at 72°C for 1 min (35 cycles), and final extension at 72°C for 2 min (1 cycle). Amplified DNA was stored at 4°C. To detect DNA bands gel electrophoresis was performed on a 3% agarose gel. After that, DNA was visualized by GelRed Nucleic Acid Gel Stain (Biotium). APP-positive DNA bands were detected via UV light at 377 bp.

### DATA DEPOSITION

Array design and raw expression files have been deposited in ArrayExpress (E-MTAB-2748, E-MTAB-2750, E-MTAB-2742, E-MTAB-2738).

## SUPPLEMENTAL MATERIAL

Supplemental material is available for this article.

## Supplementary Material

Supplemental Material
