# Supplementary material for: Generation of a neuro-specific microarray reveals novel differentially expressed noncoding RNAs in mouse models for neurodegenerative diseases
Source: RNA. 2014 Dec;20(12):1929–43. doi: 10.1261/rna.047225.114 (PMC4238357; doi:10.1261/rna.047225.114)
Supplement: Supplemental Material [file supp_20_12_1929__index.html]

Generation of a neuro-specific microarray reveals novel differentially expressed noncoding RNAs in mouse models for neurodegenerative diseases — Generation of a neuro-specific microarray reveals novel differentially expressed noncoding RNAs in mouse models for neurodegenerative diseases — Supplemental Material 

# Generation of a neuro-specific microarray reveals novel differentially expressed noncoding RNAs in mouse models for neurodegenerative diseases

## Supplemental Material

**Files in this Data Supplement:**

- Supp Figures.pdf
- Supp Table 2.xlsx
- Supp Table 1.xlsx
