# Supplementary material for: Generation of a neuro-specific microarray reveals novel differentially expressed noncoding RNAs in mouse models for neurodegenerative diseases
Source: RNA. 2014 Dec;20(12):1929–43. doi: 10.1261/rna.047225.114 (PMC4238357; doi:10.1261/rna.047225.114)

Supplementary Figure S1. ncRNA predictions included for microarray design. Heat map outlines programs of prediction, associated diseases, and associated genes for ncRNA prediction present on microarray. Number of ncRNA predictions are presented in log2 scale. The y-axis shows associated gene names on the left and disease name and the right. Prediction algorithms are indicated on top. Associations were either based on mutations or biological pathways. The term “SNPs” in ALS refers to SNP “rs6690993” and “rs10493256”, which are both located in intergenic regions. Frequency of zero is indicated in grey.

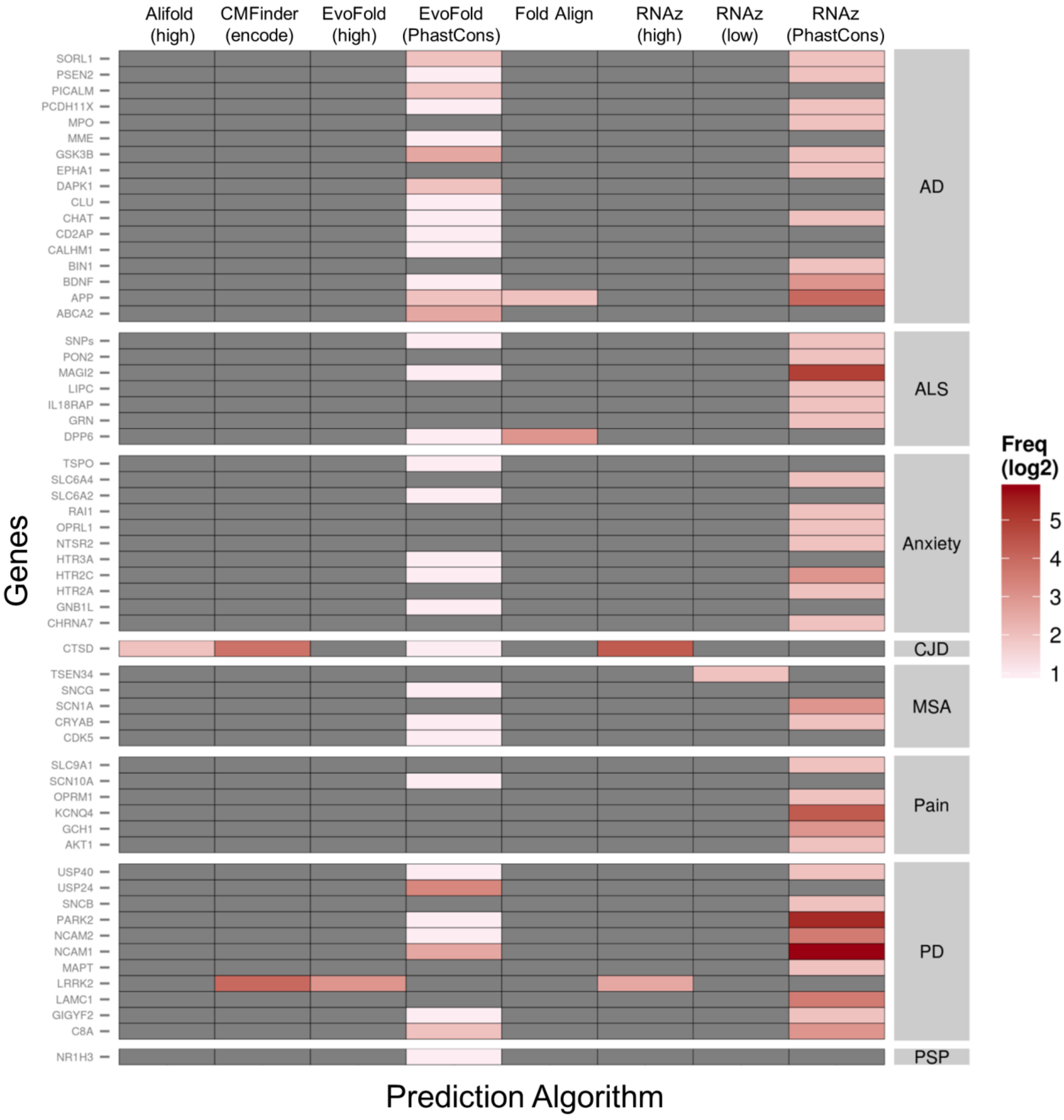

Supplementary Figure S2. Signal distribution in self-self hybridization experiments performed with RNA from whole mouse brain. MA Plot indicates fold change in a log2 scale (y-axis) and the average signal intensity (x-axis) of ncRNA candidates. Multiple testing corrected p-values showed a minimum value of 0.99999.

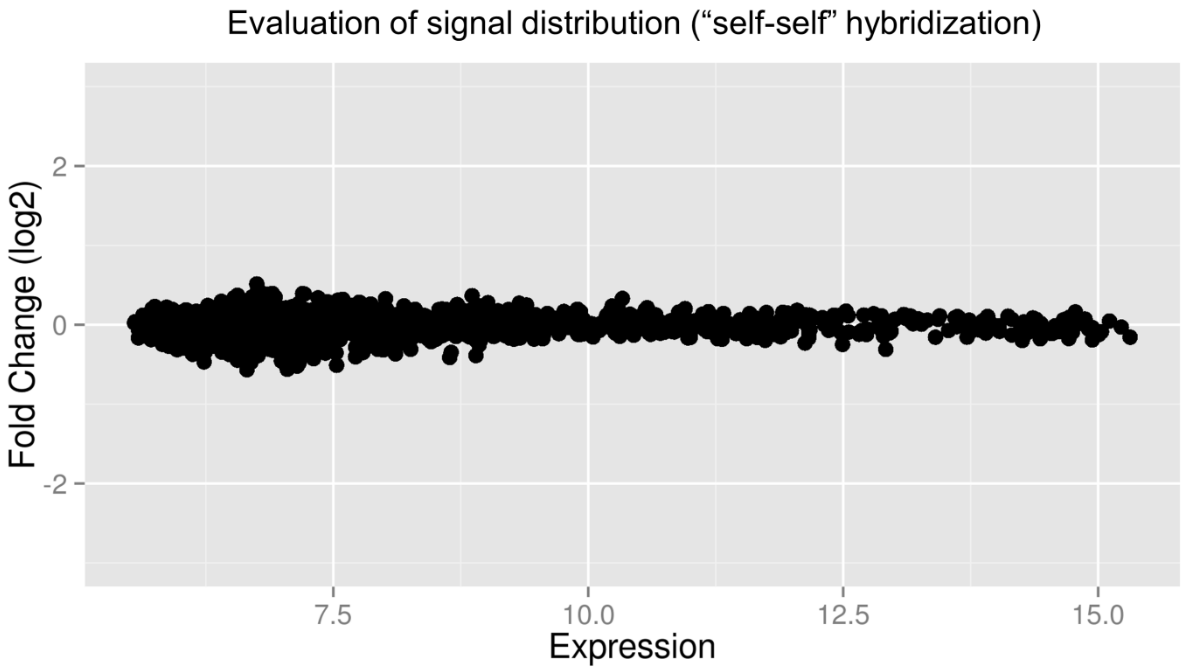

Supplementary Figure S3. tRNA-derived fragments (tRF) Northern-blot analysis of candidate e290 **(a)** and e388 **(b)** with 20 µg of whole mouse brain RNA. Snapshots of UCSC Genome Browser Views for e290 **(c)** and e388 **(d)**. Sequencing reads are represented as coverage tracks in red. tRNA-Leu-AAG and tRNA-Cys-GCA are indicated in blue as predicted by tRNAscan-SE software, respectively. The positions of oligonucleotides used for the detection of e290 and e388 are indicated in black.

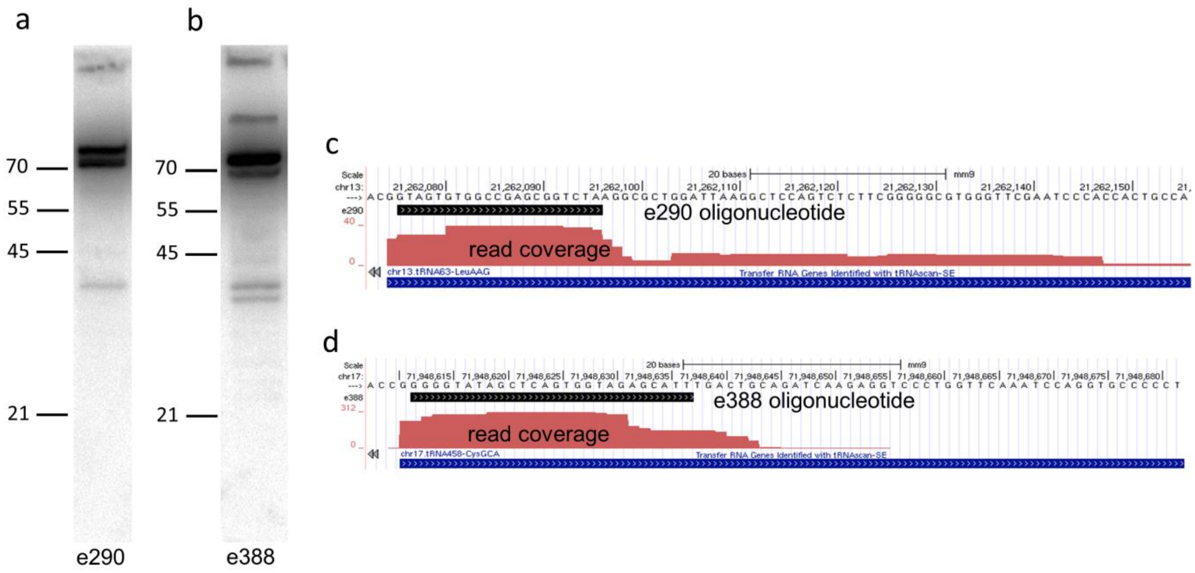

Supplement: Supplemental Material [file supp_047225.114_Supp_figures.pdf]
